# Supplementary material for: Saccharomyces boulardii (CNCM I-745) improves intestinal damage in sepsis by remodeling intestinal flora structure
Source: Front Cell Infect Microbiol. 2025 Jul 31;15:1602792. doi: 10.3389/fcimb.2025.1602792 (PMC12350329; doi:10.3389/fcimb.2025.1602792)
Supplement: Supplementary file 1 [file DataSheet1.zip › summary/summary/8_Advanced_analyse/2_PICRUSt2_result/KEGG/Group.DW4_vs_CW4_vs_BW4_vs_AW4/PICRUSt2_KEGG_level3_Group.DW4_vs_CW4_vs_BW4_vs_AW4_diff.pdf]

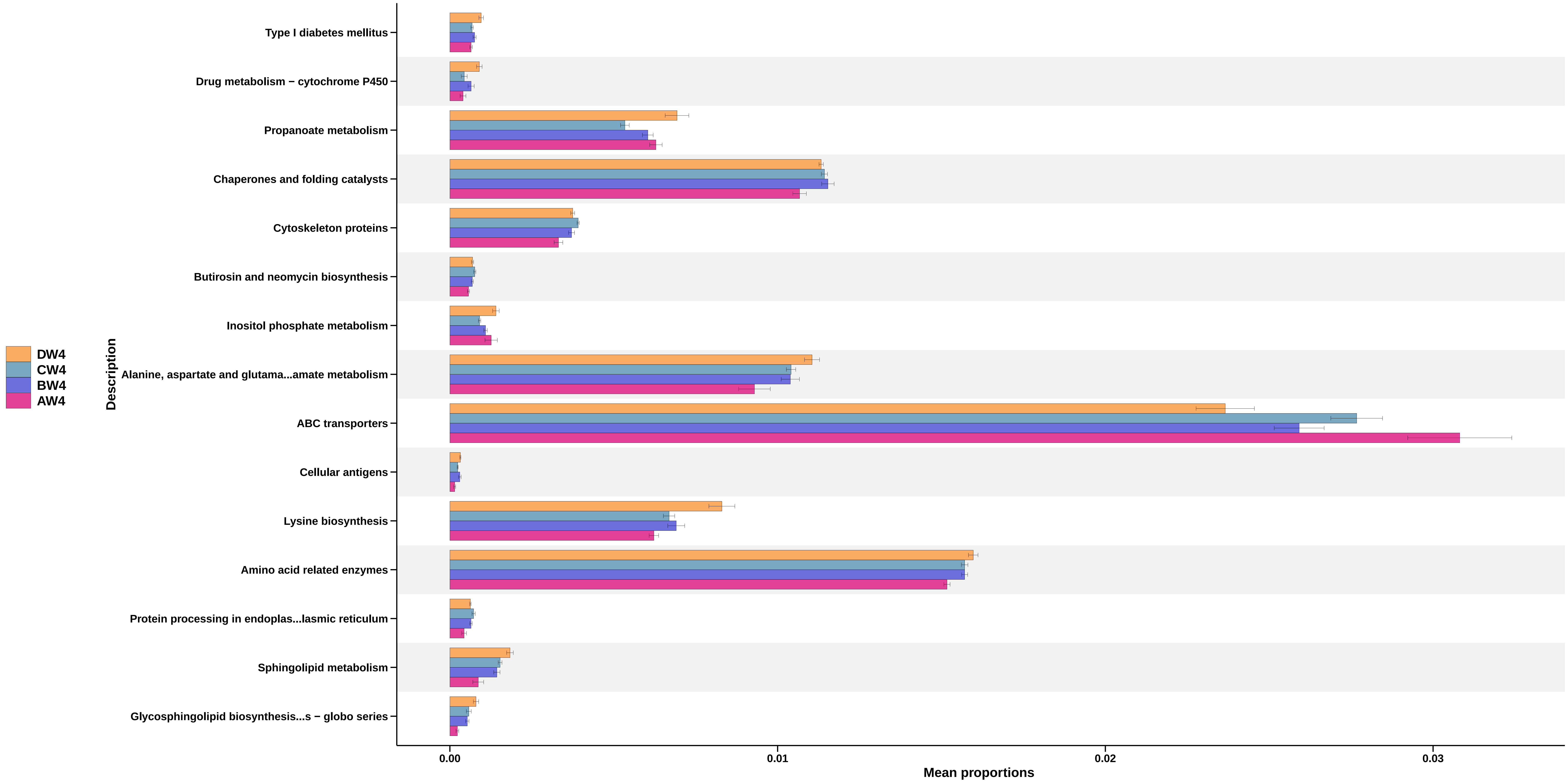
DW4
CW4
BW4
AW4
Description
Type I diabetes mellitus
Drug metabolism − cytochrome P450
Propanoate metabolism
Chaperones and folding catalysts
Cytoskeleton proteins
Butirosin and neomycin biosynthesis
Inositol phosphate metabolism
Alanine, aspartate and glutama...amate metabolism
ABC transporters
Cellular antigens
Lysine biosynthesis
Amino acid related enzymes
Protein processing in endoplas...lasmic reticulum
Sphingolipid metabolism
Glycosphingolipid biosynthesis...s − globo series
0.00
0.01
0.02
0.03
Mean proportions
